# Performance of the new clinical case definitions of pertussis in pertussis suspected infection and other diagnoses similar to pertussis

**DOI:** 10.1371/journal.pone.0204103

**Published:** 2018-09-20

**Authors:** Mioljub Ristić, Biljana Radosavljević, Vesna D. Stojanović, Milan Đilas, Vladimir Petrović

**Affiliations:** 1 Department of Epidemiology, Faculty of Medicine, University of Novi Sad, Novi Sad, Serbia; 2 Centre for Disease Control and Prevention, Institute of Public Health of Vojvodina, Novi Sad, Serbia; 3 Centre for Microbiology, Institute of Public Health of Vojvodina, Novi Sad, Serbia; 4 Department of Paediatrics, Faculty of Medicine, University of Novi Sad, Novi Sad, Serbia; 5 Institute for Child and Youth Health Care of Vojvodina, Novi Sad, Serbia; Kliniken der Stadt Köln gGmbH, GERMANY

## Abstract

**Background:**

In an effort to improve the pertussis diagnosis, the Global Pertussis Initiative (GPI) proposed an algorithm of the signs/symptoms of pertussis for three age groups: 0–3 months, 4 months to 9 years, and ≥10 years of age.

**Methods:**

We evaluated the accuracy of the clinical case definitions for pertussis proposed by the GPI using laboratory-confirmed pertussis as a reference standard for four groups: clinically suspected pertussis without comorbidity; asthma exacerbation; allergic constitution, and other diagnoses (bronchitis, bronchiolitis, laryngitis, and tracheitis). We included only patients who fulfilled one or more criteria of clinical case definitions for the age groups (0–3 months, 4 months–9 years, and ≥10 years of age).

The data for this prospective epidemiological study were collected between 1^st^ January 2013–31^st^ December 2016 at the outpatients and inpatients health care settings in the South Bačka District of Autonomous Province of Vojvodina, Serbia. We evaluated accuracy of the certain sign and symptom combinations of GPI case definitions based on their sensitivity, specificity, and likelihood ratios.

**Results:**

A total of 1043 participants were included, with 306 (29.3%) laboratory-confirmed pertussis cases. In patients aged 0–3 months, whoop and apnoea associated with laboratory confirmation of pertussis. In patients aged 4 months-9 years with a pertussis suspicion infection or with one of the other diagnoses, the highest accuracy was found for whoop combined with apnoea or post-tussive emesis. In patients aged 10 years and older, several different sign and symptom combinations were associated with an increased risk of pertussis among all enrolment diagnoses. There were fewer hospitalizations among the fully vaccinated children than in partly or unvaccinated children aged 4 months to 6 years (20.7% vs. 60.0%, p = 0.017).

**Conclusions:**

The numerous sign and symptom combinations in the observed case definitions were good predictors for laboratory-confirmed pertussis among all enrolment diagnoses, therefore suggesting the necessity for increased awareness of possibility for pertussis in patients with certain pertussis-like medical conditions.

## Introduction

Pertussis is a highly contagious respiratory illness and a major cause of infant morbidity and mortality worldwide [1, 2]. Due to many different existing case definitions throughout the world, heterogeneity of clinical manifestations, the change of clinical features induced by immunization, and mixed infections, pertussis still represent an under-estimated disease worldwide [3–5].

Many medical conditions (non-infectious diseases or various infections) can resemble pertussis. Among the common causes of prolonged cough are asthma, gastroesophageal reflux disease, and the allergies (atopic constitution). The paroxysmal cough in the laboratory-confirmed pertussis can appear more severe than expected in a patient with one of the above mentioned medical conditions. Furthermore, subacute cough is a common sign/symptom of upper and lower respiratory tract infection [6].

With the aim of unifying surveillance of pertussis, the Global Pertussis Initiative (GPI) proposed an algorithm for the signs and symptoms of pertussis in three age groups: 0–3 months, 4 months to 9 years, and ≥10 years of age [5].

The surveillance of pertussis has been conducted in the Autonomous Province of Vojvodina (APV)—the northern region of Serbia with a population of 1,931,809—since 1948. However, until September 15th, 2012, pertussis in our region was only reported according to the clinical diagnosis, without a specific clinical case definition or laboratory confirmation [7, 8].

In Serbia, the routine vaccination against pertussis with whole cell pertussis vaccine (DTwP) started in 1960, and in 2015, the DTwP vaccine has been replaced by the DTaP-IPV-Hib (combined diphtheria—tetanus—pertussis—polio—Hib vaccine). Since 2004, a portion of children population were vaccinated by the DTaP-IPV-Hib, available in private markets [9].

The aim of the present study was to evaluate the diagnostic value of certain sign and symptom combinations from case definitions of pertussis proposed by the GPI among patients with clinically suspected pertussis without comorbidity, or with suspected pertussis infection accompanied by the one of the following diagnoses (exacerbation of asthma, allergic constitution, bronchitis, bronchiolitis, laryngitis, and tracheitis). Additionally, we determined the frequency of certain sign and symptom combinations and risk of hospitalization according to the documented vaccination status of the participants.

The findings of this study provide an insight into usefulness of the case definition proposed by the GPI and point to the necessity of raising awareness among clinicians, especially during examination of pertussis-like medical conditions.

## Materials and methods

### Study design

A retrospective analysis of prospectively collected data, at the primary (outpatients) and secondary or/and tertiary (inpatients) health care settings in the South Bačka District (SBD) of the APV, was conducted. The SBD is one of seven administrative districts of the APV. According to the 2011 census results, it has a population of 615.371 inhabitants, which represents 32% of the total APV population.

The participants were enrolled by 11 Health Centres (primary health care level) of SBD and by Clinical Centre of Vojvodina, Novi Sad, Department of Pulmology, Institute for Child and Youth Health Care of Vojvodina, Institute of Pulmonary Diseases of Vojvodina and the General Hospital of Vrbas (inpatients facilities), from the 1 ^st^ January, 2013 to the 31 ^st^ December, 2016. The education about pertussis disease, the proposed case definitions of pertussis, as well as the adequate sampling and the samples handling procedures of the all included physicians and nurses was conducted before starting of the research and lasted one month.

### Inclusion/Exclusion criteria

**Fig 1:**
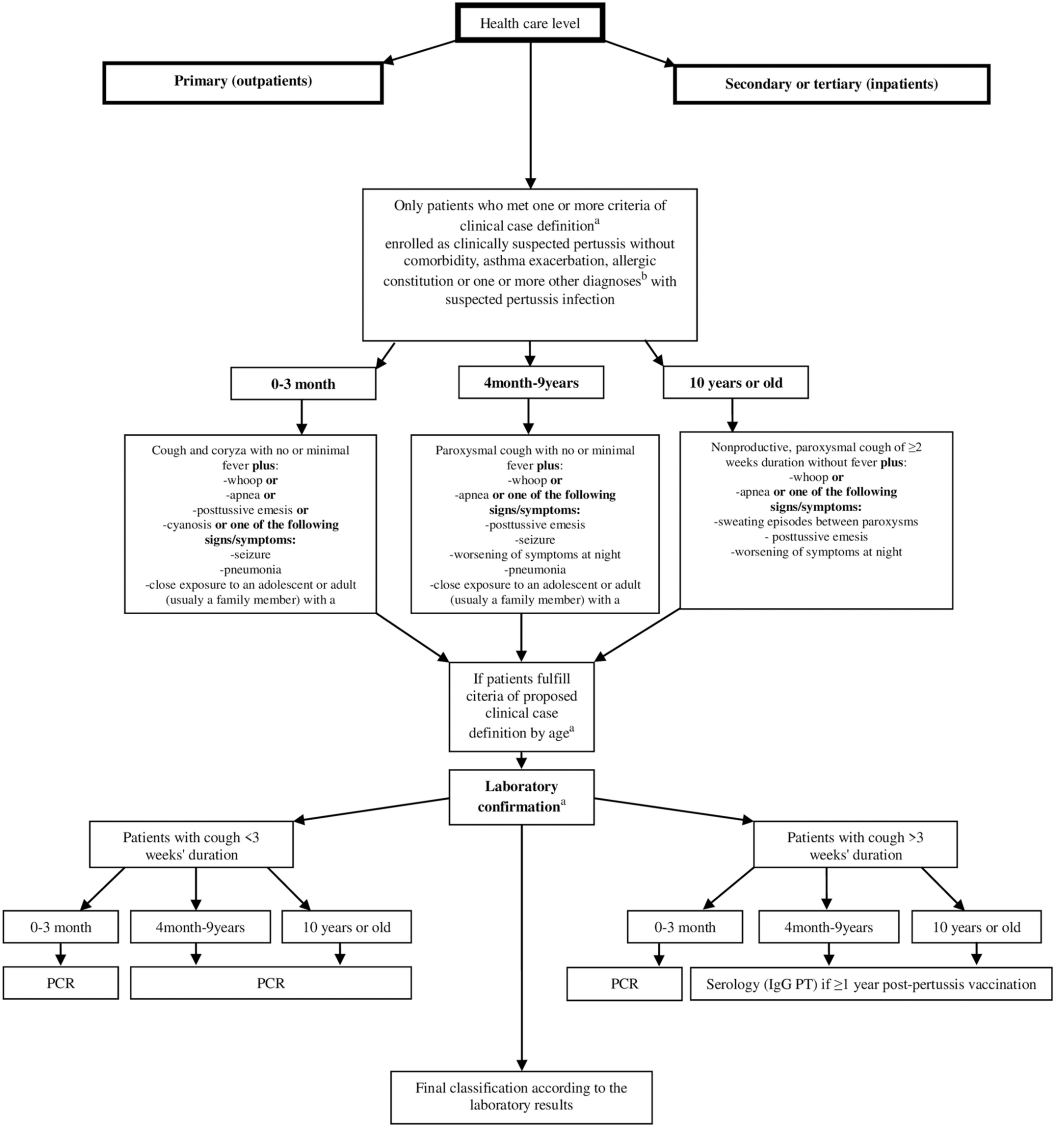
Algorithm for the surveillance and laboratory diagnosis of pertussis. ^a^ Clinical case definition and procedures for laboratory confirmation in accordance with the recommendations of the Global Pertussis Initiative (GPI) (Cherry at all, Clinical Definitions of Pertussis: Summary of a Global Pertussis Initiative Roundtable Meeting, February 2011. Clinical Infectious Diseases Advance Access published May 4, 2012); ^b^ Other diagnoses included bronchitis, bronchiolitis, laryngitis or tracheitis. PCR: polymerase chain reaction; IgG: immunoglobin G; PT: pertussis toxin.

Participants included in this study were selected and sampled by the physicians in the two health care levels as a part of the daily routine. The study design is presented on Fig 1.

We included only patients who fulfilled one or more criteria of clinical case definitions for the age groups (0–3 months, 4 months–9 years, and ≥10 years of age). Patients were excluded if they did not fulfil any of proposed clinical criteria given by GPI case definition of pertussis for the mentioned age groups. Furthermore, based on the clinical data, subjects with suspected pertussis were classified into four medical conditions as enrolment diagnosis: patients with suspected pertussis only (without any other known medical conditions previously); persons with recent exacerbation of asthma; respondents with previously confirmed allergic constitution and with symptoms of respiratory infection during admission, and patients who had one or more of the following diagnoses: bronchitis, bronchiolitis, laryngitis, tracheitis, at the first medical visit. Participants with all enrolment diagnoses were grouped according to the diagnoses given by physicians included in the research.

All the subjects, who visited one of the outpatients or inpatients health care settings, and who met one or more criteria of proposed clinical case definition of pertussis according to the age were interviewed by physicians at the admission.

Information about the suspicious pertussis cases were collected through face-to-face structured interviews. The questionnaire was comprised of questions about on sociodemographic features and vaccination against pertussis, as well as of questions related to the date of onset of the cough and questions about all clinical signs and symptoms of pertussis divided in the three age groups and enrolment diagnoses (Fig 1, S1 Questionnaire).

Physicians, who interviewed participants, gave additional explanation for the questions that could have been misunderstood.

### Specimen collection and laboratory testing

The transport of clinical samples from physicians to the laboratory of the Institute of Public Health of Vojvodina was organized on a daily basis in a hand refrigerator (at +2– +8 °C), together with the previously completed questionnaires. Diagnosis of pertussis was performed using real-time polymerase chain reaction (RT-PCR) and ELISA antibody tests. Posterior nasopharyngeal (PNP) swabs, for RT-PCR and whole blood (WB) samples, for ELISA assays, were collected by trained study physicians (PNP) and nurses (WB). Nasopharyngeal swabs and single-serum from patients were analysed at the Centre for Microbiology of Institute of Public Health of Vojvodina, Novi Sad. According to the pertussis case definition of the GPI [5], the type of laboratory method (RT-PCR or serology tests) depended on the duration of coughing and on the age of the suspected case (Fig 1). Extraction of DNA and RT-PCR analysis from PNP specimens was performed using Bordetella R-geneTM commercial kit (ARGENE, BioMerieux, France), by targeting a high copy of number specific IS481 sequence, present in B.pertusis, B.holmesii and B.bronchiseptica genomes. Positive samples were further analysed using B.pertussis/B. parapertussis/B. bronchiseptica RealTM commercial kit (Sacace, Italy) for detection of conservative region of single copy number target ptxA gene that codes pertussis toxin located in B.pertussis, B. parapertussis and B.bronchiseptica genomes, as well as for detection of specific regions in genomes of B.pertusis and B.bronchiseptica. RT-PCR analyses were performed on Applied Biosystems 7500 (Applied Biosystems, USA) and SaCycler-96 system (Sacace, Italy). The results were interpreted following the manufacturer’s instructions.

The laboratory confirmation of pertussis by serology tests was performed by ELISA IgA and IgG tests with the following commercial kits: Anti-Bordetella pertussis toxin ELISA (IgG) with 4 calibrators 5 IU/ml, 25 IU/ml, 100 IU/ml and 200 IU/ml; Anti-Bordetella pertussis toxin ELISA (IgA) with 4 calibrators 2 IU/ml, 10 IU/ml, 25 IU/ml and 50 IU/ml (Euroimmun, Germany).

### Laboratory classification

All participants with laboratory confirmation of pertussis (RT-PCR-positive for B. pertussis or anti-PT IgG antibody titers ≥100 IU/ml) were considered as confirmed cases (positive). Participants with RT-PCR negative results and with anti-PT IgG antibody titers < 40 IU/ml were considered negative. Among all participants with ELISA IgG test values between 40 to < 100 IU/ml, the additional serology IgA ELISA test was used in order to classify the case. According to the age-dependent reference ranges for groups (ADR), the age-specific cut-off values for laboratory confirmation of anti-PT IgA (measured in ELISA international units per millilitre—IU/ml) by the age group were: 2 IU/ml for 0–4 year olds; 6 IU/ml for 5–10 year olds; 12 IU/ml for those older than 11 years old.

Immediately after testing was finished, the results were sent by messaging system through a special communication network to all the participants of our surveillance study.

### Final classification of participants

Confirmed pertussis cases (positive) were defined as those patients who fulfilled one or more criteria of clinical case definitions for the age groups (0–3 months, 4 months–9 years, and ≥10 years of age), accompanied by the laboratory confirmation (RT-PCR or single-serum serology). Participants without laboratory confirmation of B. pertussis infection by RT-PCR or single-serologic tests were considered negative.

According to the vaccination status, we classified respondents as fully vaccinated according to their age, partly vaccinated (cases who received some but not all the vaccination doses required for their age), and unvaccinated. Due to waning of pertussis vaccine-induced immunity over time, vaccination status was only recorded for participants younger than 19.

Pertussis vaccination schedule in Serbia includes the first series of vaccine at the age of 2–6 months, and one toddler booster dose one year after the third primary dose. If for any reason immunization has not been completed in this time frame, it should be completed by the age of 5 [10].

### Data analysis

Parametric and non-parametric, correlative, linear and nonlinear regressive analyses were performed using SPSS software tool (version 22) MedCalc for Windows, version 12.3.0 (MedCalc Software, Mariakerke, Belgium). Different statistical analyses were performed for different variables, as appropriate: t-test and ANOVA-Kruskal–Wallis H-test for continuous variables, and Fisher exact test or chi-square for categorical data. The diagnostic value of clinical features was measured using sensitivity, specificity, positive likelihood ratio (LR+), negative likelihood ratio (LR-), accuracy (S1 Table), and 95% CI for the certain sign and symptom combinations of the GPI.

Tests of proportion were performed in order to compare sensitivities and specificities between patients with pertussis without comorbidity and three other enrolment diagnoses. Values of OR>1.1 and LR+ ≥ 2 for the certain sign and symptom combinations were considered as thresholds for the increased probability of disease. A test with an accuracy value above 0.71 was considered of useful diagnostic value [11]. Statistical significance was set at the value of p<0.05.

Since only 10 laboratory-confirmed pertussis cases were registered among the infants (aged 0–3 months), a validation of certain symptoms in this age group was not performed.

### Ethical considerations

A study was conducted as a part of the daily clinical routine practice. Oral informed consent was obtained from patients at the moment of swab taking in accordance with national regulations. In accordance with applicable laws and regulations, no clearance by an Ethics Committee for the retrospective analysis of anonymized data is required in Serbia. No authors of this study treated the patients included in the analysis, and the data were anonymized before the authors accessed it.

## Results

A total of 1043 participant were enrolled in this study between 1^st^ January 2013 and 31^st^ December 2016. Out of those patients, 306 (29.3%) were tested positive for pertussis. B. parapertussis or B. bronchiseptica were not detected in our patients. Furthermore, there were no participants with suspected pertussis, who were vaccinated against pertussis in the period of 12 months before included in the study. There were no deaths recorded.

Out of all confirmed cases, there were 203 (66.4%) patients with clinically suspected pertussis without comorbidity as enrolment diagnosis, while 103 (33.7%) participants were classified as suspected pertussis infection in combination with asthma, allergic constitution or other diagnoses similar to pertussis. Furthermore, 200 (65.4%) cases were registered at the primary health care level, while 106 (34.6%) patients were identified as inpatients.

Regarding the prevalence of all confirmed cases, participants with asthma exacerbation had the highest prevalence of laboratory confirmation (24 of 67, 35.8%), followed by participants with allergic constitution (23 of 66, 34.8%). Stratified by three age groups, prevalence of laboratory-confirmed pertussis was the highest in the patients aged 4 months to 9 years old who had pertussis without comorbidity or asthma or allergic constitution, while in the participants with other diagnoses the highest prevalence of pertussis was detected among infants aged 0–3 months.

Comparing the prevalence of laboratory-confirmation of pertussis among participants with suspected pertussis without comorbidity, it was significantly higher inpatients (67 of 157, 42.7%) than outpatients (136 of 505, 26.9%) (p<0.001). Furthermore, prevalence of laboratory-confirmation in inpatients (41 of 95, 43.2%) aged 10 years old and older with suspected pertussis without any comorbidity was significantly higher than among outpatients (76 of 302, 25.2%) (p<0.001). Although the prevalence of confirmed pertussis cases in the respondents with other three enrolment diagnoses was higher in inpatients than outpatients, the differences were not statistically significant (p>0.05) (Table 1).

**Table 1:** Diagnosis at enrollment of the 1043 patients tested for Bordetella pertussis.

Diagnosis at enrolment	Total(n = 1043)			Outpatients(n = 775)			Inpatients(n = 268)			p value ^a^
	Positive	Negative	Prevalence of confirmed cases (%)	Positive	Negative	Prevalence of confirmed cases (%)	Positive	Negative	Prevalence of confirmed cases (%)	
	(n = 306)	(n = 737)		(n = 200)	(n = 575)		(n = 106)	(n = 162)		
	n (%)	n (%)		n (%)	n (%)		n (%)	n (%)		
All clinically suspected pertussis without comorbidity	203(66.4)	459(62.3)	30.7	136(68.0)	369(64.2)	26.9	67(63.2)	90(55.6)	42.7	**<0.001**
	n (%)	n (%)		n (%)	n (%)		n (%)	n (%)		
0–3 months	5(2.5)	12(2.6)	29.4	1(0.7)	4(1.1)	20.0	4(6.0)	8(8.9)	33.3	1.000 ^b^
4m-9 years	81(39.9)	167(36.4)	32.7	59(43.4)	139(37.7)	29.8	22(32.8)	28(31.1)	44.0	0.056
10 years and older	117(57.6)	280(61.0)	29.5	76(55.9)	226(61.2)	25.2	41(61.2)	54(60.0)	43.2	**<0.001**
All suspected pertussis and confirmed asthma	24(7.8)	43(5.8)	35.8	13(6.5)	29(5.0)	31.0	11(10.4)	14(8.6)	44.0	0.281
	n (%)	n (%)		n (%)	n (%)		n (%)	n (%)		
0–3 months	0(-)	0(-)	-	-	-	-	-	-	-	-
4m-9 years	9(37.5)	6(14.0)	60.0	5(38.5)	3(10.3)	62.5	4(36.4)	3(21.4)	57.1	1.000 ^b^
10 years and older	15(62.5)	37(86.0)	28.8	8(61.5)	26(89.7)	23.5	7(63.6)	11(78.6)	38.8	0.245
All suspected pertussis and confirmed allergic constitution	23(7.5)	43(5.8)	34.8	15(7.5)	31(5.4)	32.6	8(7.5)	12(7.4)	40.0	0.563
	n (%)	n (%)		n (%)	n (%)		n (%)	n (%)		
0–3 months	0(-)	0(-)	-	-	-	-	-	-	-	-
4m-9 years	6(26.1)	9(20.9)	40.0	5(33.3)	4(12.9)	55.6	1(12.5)	5(41.7)	16.7	0.287 ^b^
10 years and older	17(73.9)	34(79.1)	33.3	10(66.7)	27(87.1)	27.0	7(87.5)	7(58.3)	50.0	0.120
All suspected pertussis with other diagnoses ^c^	56(18.3)	192(26.1)	22.6	36(18.0)	146(25.4)	19.8	20(18.9)	46(28.4)	30.3	0.080
	n (%)	n (%)		n (%)	n (%)		n (%)	n (%)		
0–3 months	5(8.9)	11(5.7)	31.3	2(5.6)	6(4.1)	25.0	3(15.0)	5(10.9)	37.5	0.641 ^b^
4m-9 years	21(37.5)	75(39.1)	21.9	11(30.6)	49(33.6)	18.3	10(50.0)	26(56.5)	27.8	0.279
10 years and older	30(53.6)	106(55.2)	22.1	23(63.8)	91(62.3)	20.2	7(35.0)	15(32.6)	31.8	0.228

Values that differ significantly (p<0.05) between hospitalized and outpatients cases are marked in bold.

^a^ Differences between distribution of positive and negative pertussis cases in the outpatient and inpatients (Chi-square test).

^b^ Two-tailed Fisher’s exact test.

^c^ Other diagnoses included bronchitis, bronchiolitis, laryngitis, tracheitis.

Observed all participants, regardless of enrolment diagnosis, there were no significant differences in gender, living area and the average duration of cough between participants with positive and negative laboratory tests for pertussis (p>0.05). However, patients with positive pertussis tests were significantly younger than the group they were compared with (16.1±15.7 years vs. 22.5±22.2 years, p<0.001). Among participants with laboratory-confirmed pertussis, respondents with asthma exacerbation or those with allergic constitution had a longer duration of cough (41.5±29.7 days and 35.6±25.8 days) than the participants who had pertussis without comorbidity (29.6±19.3 days), or those with other diagnoses (26.8±18.1 days), and the differences were statistically significant (p = 0.016). These differences in duration of cough were registered among participants aged 10 years and older. Observing three common signs and symptoms for three age groups, we revealed that patients with laboratory-confirmed pertussis were more likely to have whoop, apnoea, and post-tussive emesis, and they were more frequently detected in the hospital facilities (p<0.05). Regarding the four enrolment diagnoses, there were no significant differences in frequency of post-tussive emesis, and in the probability of hospitalization among pertussis positive subjects (p>0.05). Whoop was more frequently registered (p = 0.001) among patients with pertussis positive test accompanied with allergic constitution or other diagnoses (78.3% and 73.2%), than among participants who had pertussis without any comorbidity (48.8%) or in respondents with exacerbation of asthma (45.8%). Apnoea was more frequently registered (p = 0.003) among patients with other diagnoses (30.4%), compared to other three enrolment clinical diagnoses (10.3%, 12.5% and 17.4%) (Table 2).

**Table 2:** Demographic and clinical characteristics of participants.

Characteristics	Pertussis in 1043 patients			Four clinical diagnoses in 306 patients with laboratory evidence of pertussis				
	Pertussis positive	Pertussis negative	P value	Clinically suspected pertussis without comorbidity	Asthma and pertussis	Allergic constitution and pertussis	Other diagnoses a and pertussis	p value b
	(n = 306)	(n = 737)		(n = 203)	(n = 24)	(n = 23)	(n = 56)	
	n/ N	n/ N		n/ N	n/ N	n/ N	n/ N	
	(%)	(%)		(%)	(%)	(%)	(%)	
Age (years) ^c^	16.1±15.7	22.5±22.0	**<0.001** ^d^	15.6±14.3	18.0±19.2	15.7±13.2	16.9±19.0	0.868 ^e^
Female	166	429	0.239	116	11	8	31	0.179
	(54.2)	(58.2)		(57.1)	(45.8)	(34.8)	(55.4)	
Male	140	308		87	13	15	25	
	(45.8)	(41.8)		(42.9)	(54.2)	(65.2)	(44.6)	
Urban area	226	564	0.360	150	18	16	42	0.964
	(73.9)	(76.5)		(73.9)	(75.0)	(69.6)	(75.0)	
Rural area	80	173		53	6	7	14	
	(26.1)	(23.5)		(26.1)	(25.0)	(30.4)	(25.0)	
Total average duration of cough (days) ^c^	30.4±20.9	32.6±29.5	0.236 ^d^	29.6±19.3	41.5±29.7	35.6±25.8	26.8±18.1	**0.016** ^e^
0–3 months	13.3±8.6	10.3±7.6	0.320 ^d^	14.0±5.8	-	-	12.6±9.8	0.790 ^d^
4m-9 years	25.8±17.6	27.7±28.1	0.501 ^d^	25.8±17.9	22.6±17.1	21.7±6.5	28.5±18.1	0.771 ^e^
10 years and older	34.4±22.3	36.7±30.9	0.365 ^d^	32.9±19.6	52.9±29.9	40.5±28.1	27.9±18.1	**0.001** ^e^
Total whoop	169/306	170/737	**<0.001**	99/203	11/24	18/23	41/56	**0.001**
	(55.2)	(23.1)		(48.8)	(45.8)	(78.3)	(73.2)	
	n/ N	n/ N		n/ N	n/ N	n/ N	n/ N	
	(%)	(%)		(%)	(%)	(%)	(%)	
0–3 months	8/10	5/23	**0.005**	5/5	0/0	0/0	3/5	0.444
	(80.0)	(21.7)		(100.0)	(-)	(-)	(60.0)	
4m-9 years	67/117	72/257	**<0.001**	42/81	5/9	4/6	16/21	0.225
	(57.3)	(28.0)		(51.9)	(55.6)	(66.7)	(76.2)	
10 years and older	94/179	93/457	**<0.001**	52/117	6/15	14/17	22/30	**0.001**
	(52.5)	(20.4)		(44.4)	(40.0)	(82.4)	(73.3)	
Total apnoea	45/306	28/737	**<0.001**	21/203	3/24	4/23	17/56	**0.003**
	(14.7)	(3.8)		(10.3)	(12.5)	(17.4)	(30.4)	
	n/ N	n/ N		n/ N	n/ N	n/ N	n/ N	
	(%)	(%)		(%)	(%)	(%)	(%)	
0–3 months	8/10	6/23	**0.007**	5/5	0/0	0/0	3/5	0.444
	(80.0)	(26.1)		(100.0)	(-)	(-)	(60.0)	
4m-9 years	24/117	17/257	**<0.001**	14/81	1/9	2/6	7/21	0.260
	(20.5)	(6.6)		(17.3)	(11.1)	(33.3)	(33.3)	
10 years and older	13/179	5/457	**<0.001**	2/117	2/15	2/17	7/30	**<0.001**
	(7.3)	(1.1)		(1.7)	(13.3)	(11.8)	(23.3)	
Total post-tussive emesis	166/306	167/737	**<0.001**	108/203	14/24	13/23	31/56	0.955
	(54.2)	(22.7)		(53.2)	(58.3)	(56.5)	(55.4)	
	n/ N	n/ N		n/ N	n/ N	n/ N	n/ N	
	(%)	(%)		(%)	(%)	(%)	(%)	
0–3 months	6/10	8/23	0.257	3/5	0/0	0/0	3/5	1.000
	(60.0)	(34.8)		(60.0)	(-)	(-)	(60.0)	
4m-9 years	61/117	77/257	**<0.001**	37/81	5/9	4/6	15/21	0.169
	(52.1)	(30.0)		(45.7)	(55.6)	(66.7)	(71.4)	
10 years and older	99/179	82/457	**<0.001**	68/117	9/15	9/17	13/30	0.515
	(55.3)	(17.9)		(58.1)	(60.0)	(52.9)	(43.3)	
Total hospitalized	106/306	162/737	**<0.001**	67/203	11/24	8/23	20/56	0.660
	(34.6)	(22.0)		(33.0)	(45.8)	(34.8)	(35.7)	
	n/ N	n/ N		n/ N	n/ N	n/ N	n/ N	
	(%)	(%)		(%)	(%)	(%)	(%)	
0–3 months	7/10	13/23	0.701	4/5	0/0	0/0	3/5	0.583
	(70.0)	(56.5)		(80.0)	(-)	(-)	(60.0)	
4m-9 years	37/117	62/257	0.127	22/81	4/9	1/6	10/21	0.212
	(31.6)	(24.1)		(27.2)	(44.4)	(16.7)	(47.6)	
10 years and older	62/179	87/457	**<0.001**	41/117	7/15	7/17	7/30	0.394
	(34.6)	(19.0)		(35.0)	(46.7)	(41.2)	(23.3)	

n/N: number cases/total of cases in certain age groups. NA: not applicable. Values that differ significantly between positive and negative pertussis cases are marked in bold.

^a^ Other diagnoses included bronchitis, bronchiolitis, laryngitis, tracheitis.

^b^ P values are based on the Fisher exact or chi-square tests, as appropriate.

^c^ expressed as mean ± SD.

^d^ Student’s *t* test.

^e^ ANOVA analysis of variance/Kruskal–Wallis H test.

In the age group of 0 to 3 months, pertussis was laboratory-confirmed in 5 patients with clinically suspected pertussis without comorbidity, as enrolment diagnosis, and among 5 participants with other diagnoses. In this age group, the mandatory sign/symptom (MSS) in combination with whoop or apnoea was associated with laboratory confirmation of pertussis among patients with clinically suspected pertussis without comorbidity as an enrolment diagnosis (p = 0.003). As expected for this age group, there were no patients with asthma or allergic constitution.

In the participants 4 months to 9 years old, according to the univariate and multivariate logistic regression analyses, the MSS in combination of whoop, apnoea or post-tussive emesis was strongly associated with laboratory-confirmed pertussis in patients with clinically suspected pertussis without comorbidity enrolment diagnosis and in those with other diagnoses (p<0.05). Furthermore, among respondents with clinically suspected pertussis without comorbidity, as an enrolment diagnosis, the MSS combined with a close exposure to an adolescent or an adult with a prolonged afebrile cough illness (contact) was associated with having a laboratory-confirmed pertussis (p<0.05).

Among participants aged 10 years and older, the combination of MSS and post-tussive emesis was a good predictor of laboratory-confirmed pertussis in all four clinical diagnoses (p<0.05). Beside participants who had asthma exacerbation, the MSS in combination with whoop in this age group had a strong association with pertussis in three other diagnoses (p<0.05). Patients aged 10 years and older with other diagnoses (bronchitis, bronchiolitis, laryngitis, tracheitis) and MSS combined with apnoea were eight times more likely to have laboratory-confirmed pertussis than those without MSS and apnoea (p = 0.002).

According to the results of the univariable or a multivariable logistic regression analyses, differences among all other observed sign and symptom combinations in patients with and without pertussis infection stratified by age and clinical diagnoses, were not statistically significant (p>0.05) (S2 Table).

The diagnostic value of the selected sign and symptom combinations for pertussis in the participants aged 4 months and older, stratified in two age groups and by four enrolment diagnoses is shown in Table 3.

**Table 3:** Sensitivity, specificity, positive likelihood ratio, and negative likelihood ratio of signs and symptoms of proposed case definitions of patients older than 4 with suspected pertussis infection.

Age group with mandatory and other signs and symptoms of pertussis		Clinically suspected pertussis without comorbidity				Asthma and pertussis				Allergic constitution and pertussis				Other diagnoses ^a^ and pertussis			
		Sensitivity %	Specificity %	LR+	LR-	Sensitivity %	Specificity %	LR+	LR-	Sensitivity %	Specificity %	LR+	LR-	Sensitivity %	Specificity %	LR+	LR-
		(95% CI)	(95% CI)	(95% CI)	(95% CI)	(95% CI)	(95% CI)	(95% CI)	(95% CI)	(95% CI)	(95% CI)	(95% CI)	(95% CI)	(95% CI)	(95% CI)	(95% CI)	(95% CI)
**4m-9 years** Paroxysmal cough with no or minimal fever plus:	Whoop	51.9	74.3	**2.0**	0.7	55.6	50.0	1.1	0.9	66.7	77.8	**3.0**	0.4	76.2	68.0	**2.4**	0.4
		(40.5–63.1) ^c^	(66.9–80.7)	(1.4–2.8)	(0.5–0.8)	(21.2–86.3)	(11.8–88.2)	(0.4–3.0)	(0.3–2.6)	(22.3–95.7)	(40.0–97.2)	(0.8–11.5)	(0.1–1.4)	(52.8–91.8)	(56.2–78.3)	(1.6–3.6)	(0.2–0.8)
	Apnoea	17.3	93.4	**2.6**	0.9	11.1	100.0	NA	0.9	33.3	100.0	NA	0.7	33.3	92.0	**4.2**	0.7
		(9.8–27.3)	(88.5–96.7)	(1.3–5.5)	(0.8–1.0)	(0.3–48.3)	(54.1–100.0)		(0.7–1.1)	(4.3–77.7)	(66.4–100.0)		(0.4–1.2)	(14.6–57.0)	(83.4–97.0)	(1.6–11.1)	(0.5–1.0)
	Post-tussive emesis	45.7	72.5	1.7	0.8	55.6	50.0	1.1	0.9	66.7	77.8	**3.0**	0.4	71.4	65.3	**2.1**	0.4
		(34.6–57.1) ^c^	(65.0–79.1)	(1.2–2.3)	(0.6–0.9)	(21.2–86.3)	(11.8–88.2)	(0.4–3.0)	(0.3–2.6)	(22.3–95.7)	(40.0–97.2)	(0.8–11.5)	(0.1–1.4)	(47.8–88.7) ^c^	(53.5–76.0)	(1.4–3.1)	(0.2–0.9)
	Worsening of symptoms at night	63.0	44.3	1.1	0.8	66.7	50.0	1.3	0.7	50.0	55.6	1.1	0.9	38.1	54.7	0.8	1.1
		(51.5–73.4) ^c^	(36.6–52.1)	(0.9–1.4)	(0.6–1.2)	(29.9–92.5)	(11.8–88.2)	(0.5–3.4)	(0.2–2.3)	(11.8–88.2)	(21.2–86.3)	(0.4–3.3)	(0.3–2.4)	(18.1–61.6) ^c^	(42.8–66.2)	(0.5–1.5)	(0.8–1.7)
	Pneumonia	0	98.2	NA	1.0	-	-	-	-	-	-	-	-	4.8	90.7	0.5	1.0
		(-)	(94.8–99.6) ^c^		(0.9–1.0)									(0.1–23.8)	(81.7–96.2) ^c^	(0.1–3.9)	(0.9–1.2)
	Seizure	11.1	95.8	**2.7**	0.9	22.2	83.3	1.3	0.9	NA	88.9	NA	1.1	14.3	93.3	**2.1**	0.9
		(5.2–20.1)	(91.6–98.3)	(1.0–6.9)	(0.9–1.0)	(2.8–60.0)	(35.9–99.6)	(0.2–11.6)	(0.6–1.5)		(51.8–99.7)		(0.9–1.4)	(3.1–36.3)	(85.1–97.8)	(0.6–8.2)	(0.8–1.1)
	Contact ^b^	39.5	77.3	1.7	0.8	44.4	83.3	**2.7**	0.7	16.7	88.9	1.5	0.9	19.0	88.0	1.6	0.9
		(28.8–51.0)	(70.1–83.4)	(1.2–2.6)	(0.6–1.0)	(13.7–78.8)	(35.9–99.6)	(0.4–18.4)	(0.3–1.3)	(0.4–64.1)	(51.8–99.7)	(0.1–19.6)	(0.6–1.4)	(5.5–41.9)	(78.4–94.4)	(0.5–4.7)	(0.7–1.2)
**10 years and older** Nonproductive, paroxysmal cough of ≥2 weeks duration without fever plus:	Whoop	44.4	83.6	**2.7**	0.7	40.0	75.7	1.6	0.8	82.4	73.5	**3.1**	0.2	73.3	72.6	**2.7**	0.4
		(35.3–53.9) ^c^	(78.7–87.7) ^c^	(1.9–3.8)	(0.6–0.8)	(16.3–67.7)	(58.8–88.2)	(0.7–3.8)	(0.5–1.3)	(56.6–96.2) ^c^	(55.6–87.1)	(1.7–5.7)	(0.1–0.7)	(54.1–87.7) ^c^	(63.1–80.9) ^c^	(1.8–3.9)	(0.2–0.7)
	Apnoea	1.7	100.0	NA	1.0	13.3	100.0	NA	0.9	11.8	97.1	**4.0**	0.9	23.3	96.2	**6.2**	0.8
		(0.2–6.0) ^c^	(98.7–100.0) ^c^		(0.9–1.0)	(1.7–40.5) ^c^	(90.5–100.0)		(0.7–1.1)	(1.5–36.4) ^c^	(84.7–99.9) ^c^	(0.4–41.1)	(0.8–1.1)	(9.9–42.3) ^c^	(90.6–99.0) ^c^	(1.9–19.7)	(0.7–1.0)
	Sweating episodes between paroxysms	43.6	56.1	1.0	1.0	46.7	67.6	1.4	0.8	58.8	55.6	1.3	0.7	63.3	52.8	1.3	0.7
		(34.5–53.1)	(50.0–62.0)	(0.8–1.3)	(0.8–1.2)	(21.3–73.4)	(50.2–82.0)	(0.7–2.9)	(0.5–1.3)	(32.9–81.6)	(37.9–72.8)	(0.8–2.3)	(0.4–1.4)	(43.9–80.1)	(42.9–62.6)	(1.0–1.9)	(0.4–1.2)
	Post-tussive emesis	58.1	82.5	**3.3**	0.5	60.0	86.5	**4.4**	0.5	52.9	82.4	**3.0**	0.6	43.3	79.3	**2.1**	0.7
		(48.6–67.2)	(77.5–86.8)	(2.5–4.5)	(0.4–0.6)	(32.3–83.7)	(71.2–95.5)	(1.8–11.1)	(0.3–0.9)	(27.8–77.0)	(65.5–93.2)	(1.3–7.1)	(0.3–1.0)	(25.5–62.6)	(70.3–86.5)	(1.2–3.6)	(0.5–1.0)
	Worsening of symptoms at night	59.8	50.0	1.2	0.8	40.0	51.4	0.8	1.1	47.1	50.0	0.9	1.0	56.7	51.9	1.2	0.8
		(50.4–68.8)	(44.0–56.0)	(1.0–1.5)	(0.6–1.0)	(16.3–67.7)	(34.4–68.1)	(0.4–1.7)	(0.7–1.9)	(23.0–72.1)	(32.4–67.6)	(0.5–1.7)	(0.6–1.9)	(37.4–74.5)	(42.0–61.7)	(0.8–1.7)	(0.5–1.3)

Significant values of LR+ (≥2) are marked in bold.

^a^ Other diagnoses included bronchitis, bronchiolitis, laryngitis, tracheitis.

^b^ Close exposure to an adolescent or adult (usually a family member) with a prolonged afebrile cough illness.

^c^ Sensitivity and specificity significantly different between pertussis only compared to the other three clinical presentations in the same age group.

A combination of MSS along with the worsening of the symptoms at night had the highest sensitivity in participants with clinically suspected pertussis without comorbidity (aged 4 month-9 year and ≥ 10 year olds), and in the age group 4 months to 9 years with asthma exacerbation (63%, 59.8% and 66.7%, respectively). Observed by two age groups (4 months to 9 years and 10 years and older), the MSS in combination with whoop had the highest sensitivity in participants with allergic constitution (66.7% and 82.4%, respectively) and other diagnoses (76.2% and 73.3%, respectively). Combination of MSS along with apnoea in two age groups had the highest specificity in almost all of the enrolment diagnoses. For the participants with clinically suspected pertussis without comorbidity as an enrolment diagnosis, aged 4 monts-9 years, only MSS along with pneumonia (98.2%) and MSS combined with seizure (95.8%) had higher specificity values than a combination of MSS along with apnoea.

In participants aged 4 months-9 years, following combinations were most strongly associated with laboratory-confirmed pertussis: MSS with seizure (Positive likelihood ratio [LR+] 2.7, 95% CI 1.0–6.9) in patients with clinically suspected pertussis without comorbidity, MSS with contact (LR+ 2.7, 95% CI 0.4–18.4) in persons with asthma exacerbation, MSS with whoop or post-tussive emesis (LR+ 3.0, 95% CI 0.8–11.5) in subjects with allergic constitution, and MSS with apnoea (LR+ 4.2, 95% CI 1.6–11.1) for those with one or more of the following diagnoses: bronchitis, bronchiolitis, laryngitis, tracheitis. In patients aged 10 years and older, the combinations with the highest diagnostic accuracy were MSS with post-tussive emesis for the participants with clinically suspected pertussis without comorbidity, and asthma exacerbation (LR+ 3.3, 95% CI 2.5–4.5, and LR+ 4.4, 95% CI 1.8–11.1, respectively), combination of MSS along with apnoea for the participants with allergic constitution, and those with the other diagnoses (LR+ 4.0, 95% CI 0.4–41.1, and LR+ 6.2, 95% CI 1.9–19.7, respectively), while MSS with whoop had LR+ more than 2 in three enrolment clinical diagnoses (pertussis only, allergic constitution and other diagnoses). Regarding sensitivity and specificity in participants with clinically suspected pertussis without comorbidity compared with those in other three diagnoses, in age group 4 months to 9 years, MSS in combination with worsening of the symptoms at night was significantly more sensitive in patients with clinically suspected pertussis without comorbidity than in participants with other diagnoses (63% vs. 38.1%, p = 0.040), while MSS in combination with post-tussive emesis was significantly more sensitive in participants with other diagnoses than in patients with clinically suspected pertussis without comorbidity as enrolment diagnosis (71.4% vs. 45.7%, p = 0.037). Pneumonia in combination with MSS was registered only in two diagnoses (suspected pertussis without comorbidity and other diagnoses) in age group 4 months-9 years, and this combination was significantly more specific in patients with pertussis without comorbidity than in the patients with other diagnoses (98.2% vs. 90.7%, p = 0.007).

Among participants 10 years old and older, MSS combined with whoop had a significantly higher sensitivity in patients with allergic constitution and other diagnoses in comparison to respondents who were enrolled as suspected pertussis without comorbidity (82.4% vs. 44.4%, p = 0.004 and 73.3% vs. 44.4%, p = 0.005, respectively). Furthermore, due to the low sensitivity for MSS combined with apnoea in subjects with suspected pertussis only (without comorbidity) (1.7%), all other diagnoses had significantly higher sensitivity than suspected pertussis only as an enrolment diagnosis (p<0.05). The combination of MSS along with whoop had significantly higher specificity in patients with suspected pertussis without comorbidity than in participants with other diagnoses who had laboratory-confirmed pertussis (83.6% vs. 72.6%, p = 0.015) and MSS combined with apnoea had significantly higher specificity for participants with clinically suspected pertussis compared to those with allergic constitution (100% vs. 97.1%, p = 0.004), or compared to other diagnoses (100% vs. 96.2%, p = 0.001). There was no significant difference in sensitivity or specificity between participants with clinically suspected pertussis without comorbidity and other three enrolment diagnoses (p>0.05), after testing for other sign and symptom combinations in those two age groups.

We calculated accuracy for the four enrolment diagnoses of pertussis according to certain sign and symptom combinations of case definition for pertussis in children aged 4 months to 9 years and in participants aged 10 years and older (Table 4).

**Table 4:** Accuracy of certain sign/symptom combinations of GPI case definitions of pertussis in two age groups and different enrollment diagnoses.

Age group with mandatory and other signs and symptoms of pertussis		Clinically suspected pertussis without comorbidity Accuracy (95% CI)	Asthma and pertussis Accuracy (95% CI)	Allergic constitutionand pertussis Accuracy (95% CI)	Other diagnoses ^a^ and pertussis Accuracy (95% CI)
**4m-9 years** Paroxysmal cough with no or minimal fever plus:	Whoop	0.67	0.53	0.73	0.70
		(0.61–0.73)	(0.27–0.80)	(0.43–0.93)	(0.61–0.76)
	Apnoea	0.69	0.47	0.73	0.79
		(0.65–0.72)	(0.34–0.47)	(0.52–0.73)	(0.72–0.86)
	Post-tussive emesis	0.64	0.53	0.73	0.67
		(0.58–0.70)	(0.27–0.80)	(0.43–0.93)	(0.57–0.74)
	Worsening of symptoms at night	0.50	0.60	0.53	0.51
		(0.44–0.56)	(0.33–0.85)	(0.27–0.80)	(0.43–0.60)
	Seizure	0.68	0.47	-	0.76
		(0.65–0.71)	(0.26–0.59)		(0.72–0.82)
	Contact ^b^	0.65	0.60	0.60	0.73
		(0.59–0.71)	(0.31–0.73)	(0.47–0.73)	(0.68–0.80)
	Whoop + apnoea	0.71	0.47	0.67	0.82
		(0.68–0.73)	(0.34–0.47)	(0.54–0.67)	(0.77–0.84)
	Whoop + post-tussive emesis	0.71	0.33	0.87	0.83
		(0.67–0.75)	(0.21–0.55)	(0.58–0.87)	(0.75–0.90)
	Whoop + worsening of symptoms at night	0.69	0.53	0.67	0.80
		(0.64–0.74)	(0.27–0.67)	(0.54–0.67)	(0.73–0.86)
	Whoop + seizure	0.68	0.40	-	0.79
		(0.67–0.68)	(0.27–0.53)		(0.77–0.79)
	Whoop + contact ^b^	0.73	0.47	0.67	0.79
		(0.70–0.76)	(0.26–0.59)	(0.54–0.67)	(0.76–0.81)
	Apnoea + post-tussive emesis	0.67	-	-	0.84
		(0.65–0.70)			(0.78–0.86)
	Post-tussive emesis + worsening of symptoms at night	0.68	0.67	-	0.79
		(0.63–0.73)	(0.38–0.67)		(0.73–0.85)
	Post-tussive emesis + seizure	0.69	0.40	-	0.78
		(0.67–0.70)	(0.27–0.53)		(0.74–0.83)
	Seizure + contact ^b^	0.69	0.47	-	0.76
		(0.67–0.72)	(0.26–0.59)		(0.72–0.82)
	Whoop + apnoea + post-tussive emesis	0.69	-	0.67	0.83
		(0.67–0.70)		(0.54–0.67)	(0.78–0.83)
	Apnoea + post-tussive emesis + worsening of symptoms at night	0.68	-	0.67	0.80
		(0.66–0.70)		(0.54–0.67)	(0.77–0.80)
	Post-tussive emesis + worsening of symptoms at night + contact ^b^	0.69	0.53	-	-
		(0.67–0.69)	(0.32–0.53)		
	Whoop + post-tussive emesis + worsening of symptoms at night	0.70	0.47	0.67	0.82
		(0.66–0.73)	(0.34–0.47)	(0.54–0.67)	(0.77–0.82)
	Whoop + apnoea + post-tussive emesis + worsening of symptoms at night	0.69	-	0.69	0.80
		(0.67–0.69)		(0.67–0.69)	(0.77–0.80)
**10 years and older** Nonproductive, paroxysmal cough of ≥2 weeks duration without fever plus:	Whoop	0.72	0.65	0.77	0.73
		(0.68–0.76)	(0.53–0.78)	(0.62–0.85)	(0.65–0.79)
	Apnoea	0.71	0.75	0.69	0.80
		(0.70–0.71)	(0.69–0.75)	(0.62–0.72)	(0.75–0.84)
	Sweating episodes between paroxysms	0.52	0.62	0.57	0.55
		(0.48–0.57)	(0.49–0.75)	(0.42–0.70)	(0.48–0.62)
	Post-tussive emesis	0.75	0.79	0.73	0.71
		(0.71–0.79)	(0.66–0.89)	(0.59–0.84)	(0.64–0.78)
	Worsening of symptoms at night	0.53	0.48	0.49	0.53
		(0.48–0.57)	(0.36–0.62)	(0.35–0.63)	(0.45–0.60)
	Whoop + apnoea	0.71	0.73	0.71	0.81
		(0.70–0.71)	(0.69–0.73)	(0.64–0.71)	(0.77–0.82)
	Whoop + sweating episodes between paroxysms	0.72	0.73	0.80	0.81
		(0.69–0.74)	(0.67–0.77)	(0.67–0.87)	(0.75–0.86)
	Whoop + post-tussive emesis	0.75	0.77	0.78	0.82
		(0.72–0.77)	(0.69–0.77)	(0.68–0.78)	(0.77–0.86)
	Whoop + worsening of symptoms at night	0.75	0.71	0.77	0.77
		(0.71–0.77)	(0.68–0.75)	(0.64–0.85)	(0.70–0.83)
	Sweating episodes between paroxysms + post-tussive emesis	0.76	0.79	0.71	0.79
		(0.73–0.79)	(0.69–0.83)	(0.60–0.79)	(0.73–0.84)
	Sweating episodes between paroxysms + worsening of symptoms at night	0.69	0.69	0.65	0.75
		(0.66–0.73)	(0.63–0.77)	(0.55–0.76)	(0.68–0.82)
	Post-tussive emesis + worsening of symptoms at night	0.77	0.75	0.69	0.76
		(0.74–0.80)	(0.67–0.79)	(0.62–0.72)	(0.71–0.82)
	Whoop + apnoea + sweating episodes between paroxysms	0.71	-	0.69	0.79
		(0.70–0.71)		(0.65–0.69)	(0.77–0.79)
	Whoop + apnoea + post-tussive emesis	0.71	-	0.69	0.79
		(0.70–0.71)		(0.65–0.69)	(0.77–0.79)
	Whoop + apnoea + worsening of symptoms at night	0.71	0.73	0.69	0.79
		(0.70–0.71)	(0.69–0.73)	(0.65–0.69)	(0.76–0.80)
	Whoop + post-tussive emesis + worsening of symptoms at night	0.73	0.73	0.71	0.79
		(0.71–0.74)	(0.69–0.73)	(0.64–0.71)	(0.75–0.82)
	Sweating episodes between paroxysms + post-tussive emesis + worsening of symptoms at night	0.75	0.73	-	0.79
		(0.73–0.76)	(0.69–0.73)		(0.75–0.83)

^a^ Other diagnoses included bronchitis, bronchiolitis, laryngitis, tracheitis.

^b^ Close exposure to an adolescent or adult (usually a family member) with a prolonged afebrile cough illness.

In the respondents aged 4 months to 9 years, with clinically suspected pertussis without comorbidity, the highest accuracy values were observed for combination of MSS with whoop and contact (0.73, 95% CI 0.70–0.76), for combination of MSS with whoop and apnoea (0.71, 95% CI 0.68–0.73), and for combination of MSS accompanied by whoop and post-tussive emesis (0.71, 95% CI 0.67–0.75). Among patients with allergic constitution, the highest accuracy was registered for combination of MSS combined with whoop and post-tussive emesis (0.87, 95% CI 0.58–0.87). However, the other three sign and symptom combinations also had high accuracy in patients with allergic constitution, greater than 0.71 (MSS with whoop or apnoea or post-tussive emesis). In the respondents with other diagnoses, only three combinations (MSS with whoop or with post-tussive emesis or with worsening of symptoms at night) had poor diagnostic values (accuracy <0.71), while all other sign and symptom combinations had high diagnostic accuracy with the highest one observed for combination of MSS accompanied with apnoea and post-tussive emesis (0.84, 95% CI 0.78–0.86). Among participants with asthma exacerbation, the accuracy was poor for all of observed sign and symptom combinations in patients aged 4 months to 9 years.

In patients 10 years old and older, the following seven clinical sign and symptom combinations of pertussis (including mandatory sign/symptom in this age group) presented with good balance between sensitivity and specificity, and accuracy above 0.71: post-tussive emesis, whop and apnoea, whoop and sweating episodes between paroxysms, whoop and post-tussive emesis, whoop and worsening of the symptoms at night, sweating episodes between paroxysms and post-tussive emesis, and combination of whoop with post-tussive emesis and worsening of the symptoms at night. MSS along with worsening of symptoms at night showed poor diagnostic value for all the enrolment diagnoses.

Signs and symptoms of pertussis according to the GPI case definition and hospitalization data according to the age groups and vaccination status (fully vaccinated vs. partly or unvaccinated) are presented in S3 Table.

## Discussion

To the best of our knowledge, this is the first prospective, multicentre study that assessed the association of the certain sign and symptom combinations of GPI case definition in different age groups and certain pertussis-like illness.

Recognition of pertussis infection in the primary or inpatients health care facilities can be difficult, especially in patients with clinical signs and symptoms that are similar to pertussis. The main goal of our study was to evaluate prediction values of various sign and symptom combinations of GPI case definition in the patients with suspected pertussis infection in four different enrolment diagnoses. Among children, the most common causes of acute cough are respiratory tract infections [12], while recurrent infection is the most frequent cause of prolonged (subacute or chronic) cough [13]. In addition, exposures to allergens (allergic constitution) may cause coughing, too [14]. Although the cough can be a symptom in various medical conditions, the acute onset of paroxysms and severity of the coughing episodes increase the probability of laboratory-confirmed pertussis [6, 15]. Our results showed that 36% of patients with exacerbation of asthma who fulfilled proposed criteria of case definition of pertussis had a laboratory confirmation of pertussis, with the highest prevalence among the enrolment diagnoses. Furthermore, in patients with allergic constitution, the prevalence of confirmed pertussis was high (35%), while every third patient with pertussis suspected without comorbidity had a laboratory confirmation of pertussis.

Recently published data showed that prevalence of asthma in all patients with suspicion of pertussis ranged from 33% to 38% [16–18].

We found that the duration of cough was longer among participants with laboratory-confirmed pertussis who had asthma exacerbation or allergic constitution, than in subjects with pertussis without comorbidity or in those with one of the following enrolment diagnoses: bronchitis, bronchiolitis, laryngitis, tracheitis. We believe this is due to the fact that patients with asthma or allergic constitution delayed the visit to physician or due to physicians’ failure to recognize the worsening of cough indicating pertussis. Contrary to the clinical case definitions of pertussis recommended by the World Health Organization (WHO), the US Centers for Disease Control Prevention (CDC) or the European Centre for Disease Prevention and Control (ECDC), requiring the duration of cough for at least 2 weeks for all ages, in the clinical case definitions of pertussis proposed by the GPI, cough duration is dependent on the age of patient [5]. Thus, we believe that clinical case definitions proposed by the GPI are more useful for detection of pertussis, especially among participants younger than 10 years, in whom the inclusion criterion is cough of any duration. According to this, the administration of macrolides during the early stage of illness (duration of cough less than 14 days) can reduce duration and severity of symptoms and shorten the period of communicability [19]. Previous studies have reported that whoop [3], whoop or post-tussive emesis [4], whoop or post-tussive emesis or apnoea [18], post-tussive emesis or apnoea [20], whoop or post-tussive emesis or dyspnoea or cyanosis [21], were predictive signs and symptoms of laboratory-confirmed pertussis, while the results of other authors showed that the frequency of following signs and symptoms: whoop, post-tussive emesis, cyanosis, increased coughing at night, and contact with the confirmed pertussis case was similar in patients with and without established pertussis diagnosis [16].

Our findings indicate that whoop, apnoea, post-tussive emesis and worsening of symptoms at night in combination with MSS in certain age groups are associated with an increased risk of pertussis in all participants. In this study, several signs and symptoms were associated with an increased risk of pertussis, and remained significant after adjusting for potential covariates and confounders.

In the 0–3 months participants, cough and coryza with no or minimal fever (the mandatory symptom for this age group) in combination with whoop or apnoea were strongly associated with laboratory-confirmed pertussis. In the age group of 4 months to 9 years, MSS in combination with whoop or apnoea or post-tussive emesis increased the probability of laboratory-confirmed pertussis in respondents with clinically suspected pertussis without comorbidity or in those with other diagnoses (bronchitis, bronchiolitis, laryngitis, tracheitis). For participants aged 10 years and older, MSS combined with post-tussive emesis was a strong predictor of pertussis infection in all four enrolment diagnoses, while MSS with whoop was not a significant predictor of laboratory-confirmed pertussis only in those who had asthma exacerbation as enrolment diagnosis. A possible explanation for this may lie in the fact that certain physicians underrecognized whoop in patients presenting with an asthma attack. Our results strongly suggest that several sign and symptom combinations in two age groups (aged 4 month-9 year and ≥ 10 year olds) had different sensitivity and specificity values. Surprisingly, MSS along with post-tussive emesis in the patients aged 4 months to 9 years had higher sensitivity in participants with other diagnoses (bronchitis, bronchiolitis, laryngitis, tracheitis) than in those who had pertussis without comorbidity as an enrolment diagnosis. It is a common knowledge that the catarrhal stage of pertussis is similar to the common cold or to one of the above mentioned diagnoses [2]. Due to the fact that many of physicians failed to recognize this stage of illness, we believe that some of respondents ended up misclassified as other diagnoses (bronchitis, bronchiolitis, laryngitis, tracheitis) instead of clinically suspected pertussis without comorbidity. Additionally, the proposed GPI case definition for participants aged 4 months to 9 years requires paroxysmal cough with no or minimal fever, which is a commonly registered clinical feature in other above listed diagnoses. Nevertheless, it is important to emphasize that the combination of MSS along with whoop in patients aged 10 years and older had higher sensitivity in those with allergic constitution and with other diagnoses than in those with pertussis suspected without comorbidity as an enrolment diagnosis. The explanation for this remains unclear and should be addressed in the future research.

One of the main goals of this study was to identify the specific sign and symptom combinations of the GPI case definitions with high diagnostic value among four enrolment diagnoses. According to our results of accuracy, it was obvious that the worsening of symptoms at night in combination with MSS in the age groups 4 months to 9 years and 10 years and older was not associated with the increased risk of pertussis among all enrolment diagnoses. In addition, the MSS with sweating episodes between paroxysms in patients aged 10 years and older did not have high diagnostic value for laboratory-confirmed pertussis in all four diagnoses. However, it is noteworthy that the mentioned combinations with poor diagnostic values can be very useful when combined with other signs and symptoms from the proposed case definitions. In support of this, we observed that worsening of symptoms at night in combination with MSS and whoop or post-tussive emesis or apnoea in the age group of 4 months to 9 years, had high diagnostic value in patients with other diagnoses. Additionally, worsening of symptoms at night in combination with MSS, whoop and post-tussive emesis in the age group of 10 years and older had high predicting value in all four enrolment diagnoses.

It is a known fact that pertussis is frequently unrecognized in adults [1–3]. Several different sign and symptom combinations from the proposed case definition in patients aged 10 years and older improved the diagnostic utility for laboratory-confirmed pertussis. Capili CR. at al. [17] reported a potential epidemiologic association between asthma and risk of pertussis in the paediatric population. According to our results, the proposed clinical case definition of pertussis in respondents with asthma exacerbation aged 4 months to 9 years had a poor diagnostic value, but it was a good predictor of pertussis in participants aged 10 years and older, leading to the better recognition of pertussis among elderly with asthma.

Although our results showed that apnoea or post-tussive emesis in combination with MSS in a certain age group were more frequently registered in partly or unvaccinated participants younger than 19, numerous observed signs and symptoms had a similar distribution among the fully vs. partly or unvaccinated participants. Therefore, our results are in a good agreement with the fact that protection after the natural disease or due to vaccine-induced immunity to pertussis waned over time [22–26]. A Swiss study [21] that evaluated certain signs and symptoms proposed by WHO case definition of pertussis, and found that only 23% of the participants aged 3 months to 19 years with a known vaccination status were fully vaccinated (with 4 or 5 doses of the vaccine) against pertussis. The authors of this study did not determine statistically significant difference between the participants with post-tussive emesis who were unvaccinated or vaccinated with one or more dose pertussis vaccine in four age groups (>1 years, 1–4 years, 5–19 years and ≥20 years old). Our results showed that MSS with post-tussive emesis was less frequently registered in participants aged 10–14 years who were fully vaccinated (4 doses of the pertussis vaccine). Possible reasons for observed differences may be due to different inclusion and exclusion criteria, the type of diagnostic tests for laboratory confirmation, as well as due to different immunization schedules. In addition, we found that partly or unvaccinated children aged 4 months to 6 years had a greater chance of hospitalization compared with the fully vaccinated children in the same age group. Previous study [27], in which all hospitalized pertussis cases were younger than 5 years, reported that fully vaccinated pertussis cases were more rarely hospitalized than the unvaccinated ones, and partly vaccinated cases had fewer hospitalizations than unvaccinated patients. Furthermore, we registered hospitalized pertussis cases in all age groups of participants younger than 19 years. Due to the waning of vaccine-induced immunity, many participants aged 10–14 and 15–19 years were hospitalized. A possible explanation for the obvious high hospitalization rate in two mentioned age groups could lie in the fact that majority of participants were temporarily hospitalized in order to prevent spreading of the infection (chemoprophylaxis), and rarely due to complications of pertussis. Surprisingly, the MSS accompanied with worsening of symptoms at night in the youngest age group (4 months-6 years) was more frequently registered in fully vaccinated children. This unclear association could be due to the fact that some of the patients in this age group had an attack of asthma exacerbation accompanied with the worsening of symptoms at night [12, 13] as well as due to the lack of consistency among the parents for subjective symptoms of this sign/symptom.

Our study had some limitations. First, the study population involved only patients who fulfilled one or more clinical criteria for the GPI case definitions according to the age groups. Second, due to a limited number of cases who were 0–3 months old, we were unable to evaluate the performance of pertussis case definition in this age group. Third, due to the lack of consistency among the parents, some of the symptoms, such as whoop, apnoea, sweating episodes between the paroxysms, worsening of symptoms at night in children, could have biased the results of the study. Finally, since this was the first prospective study conducted in our region, it can be presumed that certain patients with suspected pertussis remained unidentified. Our questionnaire did not predict collection of the data about coverage of the patients who were previously vaccinated with whole or acellular pertussis vaccines, so this should also be taken into account when interpreting the study. Due to the limited capability, we were unable to provide further laboratory testing in the detection of aetiology (viral or bacterial) in patients with other diagnoses. Further research could provide the evaluation of the accuracy of the GPI clinical case definitions of pertussis in subjects with other medical conditions that predominantly present as a persistent cough.

In conclusion, this study demonstrates that the accuracy of clinical case definitions of pertussis in patients with suspected pertussis or in those with certain pertussis-like medical conditions varies with patient’s age and certain sign and symptom combinations. With regard to certain specificities, the proposed case definitions should be primarily used for screening purposes. The proposed case definition is especially useful for laboratory-confirmed pertussis in patients aged 10 years and older for all observed diagnoses, but not in the age group of 4 months to 9 years with asthma exacerbation. Using the proposed case definition of pertussis in different medical conditions may contribute to the early detection of pertussis cases and the immediate introduction of chemoprophylaxis that considerably influences the disease transmission among close contacts. In patients younger than 19 years, numerous clinical signs and symptoms from GPI were equally registered in patients who were fully vaccinated and those who were partly or unvaccinated against pertussis. Vaccination reduces complications such as apnoea in three age groups (4months-6 years, 10–14 years and 15–19 years old), and hospitalizations in children aged 4 months to 6 years.

## Supporting information

S1 Questionnaire (DOC)

S1 Table 2X2 table for calculation of sensitivity, specificity, positive (LR+) and negative likelihood (LR-) ratios, and accuracy. (DOC)

S2 Table Predictors of pertussis according to enrolment diagnosis and three age groups proposed for clinical case definition of pertussis. (DOCX)

S3 Table Predictors of pertussis according to vaccination status among participants with laboratory-confirmation of pertussis aged between 4 months and 19 years old. (DOCX)

## References

[1] World Health Organization. Pertussis vaccines: WHO position paper, August 2015—Recommendations. Vaccine. 2016; 34(12):1423–5. 10.1016/j.vaccine.2015.10.136 . 26562318

[2] Edwards K, Decker MD. Whooping cough vaccine In: Plotkin SA, Orenstein WA, Offit PA, editors. Vaccines. 6th ed Philadelphia: Elsevier; 2013 Pp. 447–92.

[3] Ghanaie RM, Karimi A, Sadeghi H, Esteghamti A, Falah F, Armin S, et al Sensitivity and specificity of the World Health Organization pertussis clinical case definition. Int J Infect Dis. 2010; 14: e1072–5. 20951620 10.1016/j.ijid.2010.07.005

[4] Koh MT, Liu CS, Chiu CH, Boonsawat W, Watanaveeradej V, Abdullah N, et al Under-recognized pertussis in adults from Asian countries: a cross-sectional seroprevalence study in Malaysia, Taiwan and Thailand. Epidemiol Infect. 2016; 144:1192–200. 26468043 10.1017/S0950268815002393 PMC4825214

[5] Cherry JD, Tan T, Wirsing von König CH, Forsyth KD, Thisyakorn U, Greenberg D, et al Clinical definitions of pertussis: Summary of a Global Pertussis Initiative roundtable meeting, February 2011. Clin Infect Dis. 2012; 54:1756–64. 10.1093/cid/cis302 . 22431797 PMC3357482

[6] Wessels MR, Brigham KS, DeMaria A Jr. Case records of the Massachusetts General Hospital. Case 6–2015. A 16-year-old boy with coughing spells. N Engl J Med. 2015; 372:765–73. . 25693017 10.1056/NEJMcpc1411928

[7] Institute of Public Health of Vojvodina. [Communicable diseases in Vojvodina, 2015. Annual report]. Novi Sad: Institute of Public Health of Vojvodina; 2016. Pp. 112–16. Serbian.

[8] Petrović V, Šeguljev Z, Ristić M, Radosavljević B, Đilas M, Heininger U. Pertussis incidence rates in Novi Sad (Serbia) before and during improved surveillance. Srp Arh Celok Lek. 2017;145(3–4):165–72.

[9] Heininger U, André P, Chlibek R, Kristufkova Z, Kutsar K, Mangarov A, et al Comparative Epidemiologic Characteristics of Pertussis in 10 Central and Eastern European Countries, 2000–2013. PLoS One. 2016; 11(6): e0155949 10.1371/journal.pone.0155949 . 27257822 PMC4892528

[10] Pravilnik o imunizaciji i načinu zaštite lekovima. Službeni glasnik Republike Srbije, broj 11/2006, 25/2013, 63/2013, 99/2013, 118/2013, 65/2014 i 32/2015. Serbian.

[11] Swets JA. Measuring the accuracy of diagnostic systems. Science. 1988; 240(4857):1285–93. . 3287615 10.1126/science.3287615

[12] Thompson M, Vodicka TA, Blair PS, Buckley DI, Heneghan C, Hay AD. Duration of symptoms of respiratory tract infections in children: systematic review. BMJ. 2013; 347: f7027 10.1136/bmj.f7027 . 24335668 PMC3898587

[13] Khoshoo V, Edell D, Mohnot S, Haydel R Jr, Saturno E, Kobernick A. Associated factors in children with chronic cough. Chest. 2009; 136:811–5. 10.1378/chest.09-0649 . 19567488

[14] Tarlo SM. Cough: occupational and environmental considerations: ACCP evidence-based clinical practice guidelines. Chest. 2006; 129(1 Suppl):186S–196S. 10.1378/chest.129.1_suppl.186S . 16428709

[15] Braman SS. Postinfectious Cough ACCP Evidence-Based Clinical Practice Guidelines. Chest. 2006; 129(1 Suppl):138S–146S. 10.1378/chest.129.1_suppl.138S . 16428703

[16] Lasserre A, Laurent E, Turbelin C, Hanslik T, Blanchon T, Guiso N. Pertussis incidence among adolescents and adults surveyed in general practices in the Paris area, France, May 2008 to March 2009. Euro Surveill. 2011; 16(5). pii: 19783. . 21315055

[17] Capili CR, Hettinger A, Rigelman-Hedberg N, Fink L, Boyce T, Lahr B, et al Increased risk of pertussis in patients with asthma. J Allergy Clin Immunol. 2012; 129(4):957–63. 22206778 10.1016/j.jaci.2011.11.020 PMC3321509

[18] Harnden A, Grant C, Harrison T, Perera R, Brueggemann AB, Mayon-White R, et al Whooping cough in school age children with persistent cough: prospective cohort study in primary care. BMJ. 2006; 333:174–7. 16829538 10.1136/bmj.38870.655405.AE PMC1513463

[19] Kilgore PE, Salim AM, Zervos MJ, Schmitt HJ. Pertussis: Microbiology, Disease, Treatment, and Prevention. Clin Microbiol Rev. 2016;29(3):449–86. 10.1128/CMR.00083-15 . 27029594 PMC4861987

[20] Crespo I, Cardeñosa N, Godoy P, Carmona G, Sala MR, Barrabeig I, et al Epidemiology of pertussis in a country with high vaccination coverage. Vaccine. 2011 6; 29(25):4244–8. 21496465 10.1016/j.vaccine.2011.03.065

[21] Wymann MN, Richard JL, Vidondo B, Heininger U. Prospective pertussis surveillance in Switzerland, 1991–2006. Vaccine. 2011; 29(11):2058–65. 10.1016/j.vaccine.2011.01.017 . 21251904

[22] Wearing HJ, Rohani P. Estimating the duration of pertussis immunity using epidemiological signatures. PLoS Pathog. 2009;5(10): e1000647 10.1371/journal.ppat.1000647 . 19876392 PMC2763266

[23] Lugauer S, Heininger U, Cherry JD, Stehr K. Long-term clinical effectiveness of an acellular pertussis component vaccine and a whole cell pertussis component vaccine. Eur J Pediatr. 2002; 161(3):142–6. . 11998910 10.1007/s00431-001-0893-5

[24] Torvaldsen S, Simpson JM, McIntyre PB. Effectiveness of pertussis vaccination in New South Wales, Australia, 1996–1998. Eur J Epidemiol. 2003; 18(1):63–9. . 12705625 10.1023/a:1022588118030

[25] Gustafsson L, Hessel L, Storsaeter J, Olin P. Long-term follow-up of Swedish children vaccinated with acellular pertussis vaccines at 3, 5, and 12 months of age indicates the need for a booster dose at 5 to 7 years of age. Pediatrics. 2006; 118(3):978–84. 10.1542/peds.2005-2746 . 16950988

[26] Klein NP, Bartlett J, Rowhani-Rahbar A, Fireman B, Baxter R. Waning protection after fifth dose of 600 acellular pertussis vaccine in children. N Engl J Med. 2012; 367(11):1012–9. 601 10.1056/NEJMoa1200850 . 22970945

[27] Crespo I, Toledo D, Soldevila N, Jordán I, Solano R, Castilla J, et al Characteristics of Hospitalized 551 Cases of Pertussis in Catalonia and Navarra, Two Regions in the North of Spain. PLoS One. 2015; 552 10: e0139993 10.1371/journal.pone.0139993 . 26440655 PMC4595087

