# Supplementary material for: Performance of the new clinical case definitions of pertussis in pertussis suspected infection and other diagnoses similar to pertussis
Source: PLoS One. 2018 Sep 20;13(9):e0204103. doi: 10.1371/journal.pone.0204103 (PMC6147443; doi:10.1371/journal.pone.0204103)
Supplement: S1 Questionnaire — (DOC) [file pone.0204103.s001.doc]

**INSITUTE OF PUBLIC HEALTH OF VOJVODINA**

**Novi Sad, Futoška 121**

| **S1 Questionnaire** | | | |
| --- | --- | --- | --- |
| Health Institution:  Name and surname of physician:  Physician’s mobile phone: | | | |
| **Patient (first letter of the name and first letter of the surname):** | | | |
| Birth Date: Gender: Male ❑ Female ❑ | | | |
| Address: | | | |
| Phone of the participant or parent or guardian: | | | |
| Occupation of the participant: | | | |
| Employment status: employed ❑ unemployed ❑ | | | |
| Collective: YES ❑ NO ❑, if yes, which (to write): | | | |
| Admission diagnosis: | | | |
| **Clinical data**: | | | |
| Date of onset of the cough: | | | |
| Duration of cough (days): paroxysmal cough: YES ❑ NO ❑ | | | |
| Whoop YES ❑ NO ❑ | | | |
| APNOEA YES ❑ NO ❑ CORYZA (in age younger than 3 months of age) YES ❑ NO ❑ | | | |
| Cyanosis | YES ❑ NO ❑ | Post-tussive emesis | YES ❑ NO ❑ |
| Worsening of symptoms at night | YES ❑ NO ❑ | Sweating episodes between paroxysms | YES ❑ NO ❑ |
| Pneumonia | YES ❑ NO ❑ | Seizure | YES ❑ NO ❑ |
| Close exposure to an adolescent or adult (usually a family member) with a prolonged afebrile cough illness YES ❑ NO ❑ **if YES, who (relationship)**: | | | |
| **Blood test results:** | | | |
| Leukocitosis: YES ❑ NO ❑ NOT PERFORMED ❑ Predomination of lymphocytes: YES ❑ NO ❑ NOT PERFORMED ❑ | | | |
|  | | | |
| **Does antibiotic included?**: YES ❑ NO ❑, if yes, when (date): | | | |
| First antibiotic: Days of antibiotic treatment:­­ | | | |
| Second antibiotic: Days of antibiotic treatment:­­ | | | |
|  | | | |
| **Date of specimens collected:** | | | |
| **Type of specimens**: Nasopharyngeal swab ❑ or Single-serum ❑, if single-serum than: | | | |
| Date of application the last dose of the **pertussis vaccine**_________________, unknown ❑ | | | |
| ***Clinical Case Definitions of Pertussis Proposed by the Global Pertussis Initiative:***  **For participants age group of 0 to 3 months**: **Cough** and coryza with no or minimal fever **plus:**  whoop **or**  apnoea **or**  post-tussive emesis **or**  cyanosis **or**  ONE OF THE FOLLOWING CHARACTESRISTICS:  • seizure  • pneumonia  • close exposure to an adolescent or adult (usually a family member) with a prolonged afebrile cough illness.  **For participants age group of 4 months to 9 years**: Paroxysmal **cough** with no or minimal fever **plus**:  whoop **or**  apnoea **or**  ONE OF THE FOLLOWING CHARACTESRISTICS:  • post-tussive emesis  • seizure  • worsening of symptoms at night  • pneumonia  • close exposure to an adolescent or adult (usually a family member) with a prolonged afebrile cough illness.  **For participants age group of 10 years and older**: Non-productive, paroxysmal **cough of ≥2 weeks duration** without fever **plus**:  whoop **or**  apnoea **or**  ONE OF THE FOLLOWING CHARACTESRISTICS:  • sweating episodes between paroxysms  • post-tussive emesis  • worsening of symptoms at night. | | | |

Physician: __________________________ Date: _______________
