# Supplementary material for: Performance of the new clinical case definitions of pertussis in pertussis suspected infection and other diagnoses similar to pertussis
Source: PLoS One. 2018 Sep 20;13(9):e0204103. doi: 10.1371/journal.pone.0204103 (PMC6147443; doi:10.1371/journal.pone.0204103)
Supplement: S1 Table — (DOC) [file pone.0204103.s002.doc]

**S1 Table. 2X2 table for calculation of sensitivity, specificity, positive (LR+) and negative likelihood (LR-) ratios, and accuracy**.

| Sign and symptom or Sign and symptom combinations | Laboratory test | |
| --- | --- | --- |
|  | Positive | Negative |
| Present | a | c |
| Absent | b | d |
| Total | a+b | c+d |

Sensitivity: a/(a+b); Specificity: d/(c+d); LR+: sensitivity/(1–specificity); LR-:1- sensitivity/specificity; Accuracy: (a+d)/(a+b+d+c).
