# Supplementary material for: Performance of the new clinical case definitions of pertussis in pertussis suspected infection and other diagnoses similar to pertussis
Source: PLoS One. 2018 Sep 20;13(9):e0204103. doi: 10.1371/journal.pone.0204103 (PMC6147443; doi:10.1371/journal.pone.0204103)
Supplement: S2 Table — (DOCX) [file pone.0204103.s003.docx]

**S2 Table. Predictors of pertussis according to enrolment diagnosis and three age groups proposed for clinical case definition of pertussis.**

| Age group with mandatory and other signs and symptoms of pertussis | | Clinically suspected pertussis without comorbidity | | | | Asthma and pertussis | | | | Allergic constitution and pertussis | | | | Other diagnoses ^a^ and pertussis | | | |
| --- | --- | --- | --- | --- | --- | --- | --- | --- | --- | --- | --- | --- | --- | --- | --- | --- | --- |
|  |  | crude OR  (95%CI) | p  value | adjusted  OR ^b^  (95%CI) | p  value | crude OR  (95%CI) | p  value | adjusted  OR ^b^  (95%CI) | p  value | crude OR  (95%CI) | p  value | adjusted OR ^b^  (95%CI) | p  value | crude OR  (95%CI) | p  value | adjusted OR ^b^  (95%CI) | p  value |
| 0-3 months  Cough and coryza  with no or minimal  fever plus: | Whoop | NA | **0.003** ^d^ | - | - | Not reported | | | | Not reported | | | | NA | 0.299 ^d^ | - | - |
|  | Apnoea | NA | **0.003** ^d^ | - | - |  |  |  |  |  |  |  |  | NA | 0.596 ^d^ | - | - |
|  | Post-tussive emesis | NA | 1.000 ^d^ | - | - |  |  |  |  |  |  |  |  | NA | 0.244 ^d^ | - | - |
|  | Cyanosis | NA | 0.600 ^d^ | - | - |  |  |  |  |  |  |  |  | NA | 1.000 ^d^ | - | - |
|  | Seizure | NA | 1.000 ^d^ | - | - |  |  |  |  |  |  |  |  | NA | 1.000 ^d^ | - | - |
|  | Pneumonia | NA | - | - | - |  |  |  |  |  |  |  |  | NA | 0.313 ^d^ | - | - |
|  | Contact ^c^ | NA | 0.515 ^d^ | - | - |  |  |  |  |  |  |  |  | NA | 1.000 ^d^ | - | - |
| 4m-9 years  Paroxysmal cough  with no or minimal  fever plus: | Whoop | 3.11  (1.78-5.42) | **<0.001** | 3.85  (2.05-7.24) | **<0.001** | NA | 0.833 ^d^ | - | - | NA | 0.136 ^d^ | - | - | 6.80  (2.23-20.74) | **0.001** | 5.98  (1.90-18.81) | **0.002** |
|  | Apnoea | 2.96  (1.28-6.86) | **0.011** | 11.29  (3.72-34.26) | **<0.001** | NA | 1.000 ^d^ | - | - | NA | 0.143 ^d^ | - | - | 5.75  (1.66-19.72) | **0.005** | 20.08  (3.39-118.74) | **0.001** |
|  | Post-tussive emesis | 2.21  (1.27-3.85) | **0.005** | 2.07  (1.13-3.79) | **0.019** | NA | 0.833 ^d^ | - | - | NA | 0.136 ^d^ | - | - | 4.71  (1.63-13.59) | **0.004** | 5.64  (1.84-17.32) | **0.003** |
|  | Worsening of symptoms at night | 1.35  (0.78-2.33) | 0.277 | 1.11  (0.61-2.00) | 0.735 | NA | 0.521 ^d^ | - | - | NA | 0.329 ^d^ | - | - | 0.74  (0.28-2.00) | 0.555 | 0.64  (0.23-1.80) | 0.401 |
|  | Pneumonia | NA | 0.553 | - | - | - | - | - | - | - | - | - | - | 0.49  (0.06-4.19) | 0.511 | 0.36  (0.04-3.52) | 0.379 |
|  | Seizure | 2.86  (1.02-7.97) | 0.045 | 2.07  (0.67-6.39) | 0.204 | NA | 1.000 ^d^ | - | - | NA | 1.000 ^d^ | - | - | NA | 0.367 ^d^ | - | - |
|  | Contact ^c^ | 2.22  (1.25-3.94) | **0.007** | 2.19  (1.17-4.08) | **0.014** | NA | 0.580 ^d^ | - | - | NA | 1.000 ^d^ | - | - | 1.73  (0.47-6.29) | 0.408 | 2.64  (0.64-10.87) | 0.180 |
| 10 years and older  Nonproductive, paroxysmal cough of ≥2 weeks duration without fever plus: | Whoop | 4.10  (2.51-6.59) | **<0.001** | 3.55  (2.15-5.84) | **<0.001** | 2.07  (0.58-7.44) | 0.263 | 1.90  (0.47-7.76) | 0.370 | 12.96  (3.01-55.89) | **0.001** | 7.30  (1.45-36.66) | **0.016** | 7.30  (2.92-18.23) | **<0.001** | 10.24  (3.54-29.60) | **<0.001** |
|  | Apnoea | NA | 0.086 ^d^ | - | - | NA | 0.079 ^d^ | - | - | NA | 0.255 | - | - | 7.76  (2.10-28.74) | **0.002** | 8.54  (2.19-33.32) | **0.002** |
|  | Sweating episodes between paroxysms | 0.99  (0.64-1.52) | 0.951 | 1.31  (0.82-2.08) | 0.259 | 1.82  (0.54-6.21) | 0.337 | 2.68  (0.61-11.75) | 0.191 | 1.81  (0.56-5.89) | 0.324 | 1.78  (0.42-7.53) | 0.433 | 1.93  (0.84-4.46) | 0.121 | 2.06  (0.87-4.87) | 0.100 |
|  | Post-tussive emesis | 6.54  (4.05-10.57) | **<0.001** | 6.02  (3.68-9.82) | **<0.001** | 9.60  (2.37-38.87) | **0.002** | 9.43  (2.04-43.65) | **0.004** | 5.25  (1.43-19.22) | **0.012** | 5.88  (1.16-29.66) | **0.032** | 2.92  (1.23-6.91) | **0.015** | 2.71  (1.13-6.50) | **0.025** |
|  | Worsening of symptoms at night | 1.49  (0.96-2.31) | 0.074 | 1.36  (0.86-2.13) | 0.188 | 0.70  (0.21-2.38) | 0.5717 | 0.78  (0.21-2.98) | 0.720 | 0.89  (0.28-2.85) | 0.843 | 1.46  (0.34-6.19) | 0.609 | 1.41  (0.62-3.19) | 0.409 | 1.49  (0.64-3.43) | 0.353 |

Values that differ significantly between positive and negative pertussis cases are marked in bold.

^a^ Other diagnoses included bronchitis, bronchiolitis, laryngitis, tracheitis.

^b^ Adjusted for the following variables: Age, gender, duration of cough and place (urban or rural).

^c^ Close exposure to an adolescent or adult (usually a family member) with a prolonged afebrile cough illness.

^d^ Two-tailed Fisher’s exact test.
