# Supplementary material for: Performance of the new clinical case definitions of pertussis in pertussis suspected infection and other diagnoses similar to pertussis
Source: PLoS One. 2018 Sep 20;13(9):e0204103. doi: 10.1371/journal.pone.0204103 (PMC6147443; doi:10.1371/journal.pone.0204103)
Supplement: S3 Table — (DOCX) [file pone.0204103.s004.docx]

**S3 Table. Predictors of pertussis according to vaccination status among participants with laboratory-confirmation of pertussis aged between 4 months and 19 years old.**

| Age group with required and other signs/symptoms of pertussis | Fully vaccinated  according to age  (n=198) | Partly or unvaccinated  (n=41) | p  value ^a^ |
| --- | --- | --- | --- |
| **4months-9 years**  **Paroxysmal cough with no or minimal fever plus:** | (n=99) | (n=18) |  |
| **4months-6 years** | (n=29) n (%) | (n=15) n (%) |  |
| Whoop | 19 (65.5) | 9 (60.0) | 0.751 |
| Apnoea | 6 (20.7) | 10 (66.7) | **0.006** |
| Post-tussive emesis | 13 (44.8) | 10 (66.7) | 0.213 |
| Worsening of symptoms at night | 19 (65.5) | 4 (26.7) | **0.025** |
| Pneumonia | 1 (3.4) | 0 (-) | 1.000 |
| Seizure | 3 (10.3) | 4 (26.7) | 0.207 |
| Contact ^b^ | 12 (41.4) | 6 (40.0) | 1.000 |
| Hospitalized | 6 (20.7) | 9 (60.0) | **0.017** |
| **7-9 years** | (n=70) n (%) | (n=3) n (%) |  |
| Whoop | 38 (54.3) | 1 (33.3) | 0.595 |
| Apnoea | 8 (11.4) | 0 (-) | 1.000 |
| Post-tussive emesis | 36 (51.4) | 2 (66.7) | 1.000 |
| Worsening of symptoms at night | 43 (61.4) | 2 (66.7) | 1.000 |
| Pneumonia | 0 (-) | 0 (-) | - |
| Seizure | 7 (10.0) | 0 (-) | 1.000 |
| Contact ^b^ | 22 (31.4) | 1 (33.3) | 1.000 |
| Hospitalized | 19 (27.1) | 0 (-) | 0.563 |
| **10 – 19 years**  **Nonproductive, paroxysmal cough of ≥2 weeks duration without fever plus:** | (n=99) | (n=23) |  |
| **10-14 years** | (n=77) n (%) | (n=9) n (%) |  |
| Whoop | 44 (57.1) | 6 (66.7) | 0.729 |
| Apnoea | 5 (6.5) | 3 (33.3) | **0.035** |
| Sweating episodes between paroxysms | 31 (40.3) | 4 (44.4) | 1.000 |
| Post-tussive emesis | 38 (49.4) | 9 (100.0) | **0.003** |
| Worsening of symptoms at night | 41 (53.2) | 6 (66.7) | 0.502 |
| Hospitalized | 27 (35.1) | 2 (22.2) | 0.712 |
| **15-19 years** | (n=22) n (%) | (n=14) n (%) |  |
| Whoop | 13 (59.1) | 8 (57.1) | 1.000 |
| Apnoea | 1 (4.5) | 5 (35.7) | **0.024** |
| Sweating episodes between paroxysms | 12 (54.5) | 5 (35.7) | 0.322 |
| Post-tussive emesis | 14 (63.6) | 8 (57.1) | 0.738 |
| Worsening of symptoms at night | 14 (63.6) | 8 (57.1) | 0.738 |
| Hospitalized | 6 (27.3) | 4 (28.6) | 1.000 |

Values that differ significantly between fully and partly or unvaccinated pertussis cases are marked in bold.

^a^ P values are based on the Fisher exact or chi-square tests, where appropriate.

^b^ Close exposure to an adolescent or adult (usually a family member) with a prolonged afebrile cough illness.
